# Estrogen Receptor-Mediated Effects of a Phytoestrogen-Rich Soy-Based Diet on Metabolic Health Following Ovariectomy in Aged Mice

**DOI:** 10.3390/nu18203451

**Published:** 2026-10-21

**Authors:** Terese M. Zidon, Jaume Padilla, Leighton T. McCabe, Olivia E. Stricklin, Young-Min Park, Dennis B. Lubahn, Victoria J. Vieira-Potter

**Affiliations:** 1 Division of Food, Nutrition and Exercise Sciences, College of Agriculture, Food, and Natural Resources, University of Missouri, Columbia, MO 65211, USA; tzidon@centralmethodist.edu (T.M.Z.); padillaja@missouri.edu (J.P.); leighton.t.mccabe@gmail.com (L.T.M.); stricklo@iastate.edu (O.E.S.); lubahnd@missouri.edu (D.B.L.); 2 Biology Department, Central Methodist University, Fayette, MO 65248, USA; 3 School of Medicine, University of Missouri, Columbia, MO 65211, USA; 4 Division of Health and Kinesiology, Sport Science Institute & Sports Functional Disability Institute, Incheon National University, Incheon 22012, Republic of Korea; ypark@inu.ac.kr; 5 Department of Biochemistry, College of Agriculture, Food, and Natural Resources, University of Missouri, Columbia, MO 65211, USA

**Keywords:** estrogen receptor, soy, menopause, ovariectomy, adipose tissue, energy expenditure, inflammation, metabolism, phytoestrogens, aging

## Abstract

Loss of ovarian hormones leads to obesity and insulin resistance (IR), increasing the risk of cardiovascular and metabolic diseases. We previously showed that aged female ERa knockout (αKO) and ERb knockout (βKO) mice differentially respond to ovariectomy, with a greater adverse metabolic response in βKO mice. We later showed that young ovariectomized (OVX) βKO mice were somewhat resistant to exercise-mediated metabolic benefits including adipose tissue browning. Background/Objectives: We tested the hypothesis that feeding a high phytoestrogen soy-based diet (SOY) would improve metabolic health in older OVX female mice and that αKO and βKO mice would differentially respond to the diet. Since phytoestrogens weakly bind ERs, and it is known that activation of ERa mediates insulin sensitizing and weight loss benefits, and ERb is known to improve adipocyte mitochondrial responses, we hypothesized that αKO mice would be less responsive to the systemic metabolic benefits of SOY, whereas βKO mice would be less responsive to adipocyte-specific benefits. Methods: We examined OVX and ovary-intact aged (~1 yr) wildtype (WT), αKO, and βKO female mice (*n* = 7–12/group). One week following OVX, mice were randomized to receive either SOY (585 mg phytoestrogens/kg) or a phytoestrogen-free diet (<15 mg/kg, CON) and were followed for 11 weeks. Mice were assessed for body composition (EchoMRI), energy expenditure (EE via Promethion metabolic cage system), glucose tolerance (i.p. glucose tolerance test), insulin sensitivity (HOMA-IR, Adipo-IR), adipose tissue gene and protein expression for insulin signaling markers, and fasting blood lipids. To provide additional mechanistic insight, primary adipocytes were treated with phytoestrogens and assessed for insulin-mediated phosphorylation of Akt. Results: Era mediated important metabolic benefits of SOY in aged OVX mice. OVX αKO mice were unresponsive to SOY-mediated improvements in body composition and glucose tolerance, whereas SOY improved those metrics, as well as increased spontaneous physical activity in OVX WT and βKO mice (all *p* < 0.05). Primary adipocyte cell culture experiments revealed that insulin-mediated phosphorylation of Akt occurred even with ERα-antagonization, with no effects of estrogen or phytoestrogen treatment, at least in the small set of experiments performed. Conclusions: SOY improved body composition and glucose tolerance in old OVX mice, effects that were absent in ERα-null mice. Soy-based diets may improve metabolic health in settings of ovarian hormone suppression via mechanisms requiring estrogen signaling pathway activation.

## 1. Introduction

Estrogen (17β-estradiol [E_2_]) deficiency in menopausal women and ovariectomized (OVX) rodents is associated with reduced energy expenditure (EE), metabolic disturbances (i.e., insulin resistance (IR)), increased adiposity, and white adipose tissue (WAT) dysfunction [1,2,3,4,5]. Thus, E_2_ deficiency increases the risk for developing obesity-related cardiovascular and metabolic diseases, such as type 2 diabetes [6,7]. E_2_ signaling occurs primarily via the classical genomic pathway, through nuclear receptors, estrogen receptor alpha (ERα) and beta (ERβ) [8,9]. Naturally occurring estrogens include E_1_ (estrone, a weaker estrogen which becomes the major form in postmenopausal women), E_2_ (estradiol, the most potent and prevalent estrogen in non-pregnant females during the reproductive years), E_3_ (estriol, a weaker estrogen produced by the placenta during pregnancy), and E_4_ (estestrol, a fetal estrogen produced exclusively during pregnancy). E_1_ and E_2_ are present in both sexes, yet E_2_ is the primary sex hormone in females and thus present at much greater levels in premenopausal females compared to age-matched males. In addition to naturally occurring estrogens, plant estrogens (i.e., phytoestrogens) exist in a variety of foods, most notably soy. Estrogen-like compounds are also found in nature in a variety of industrial and consumer chemicals (e.g., Bisphenol A). We previously demonstrated that a soy protein-based diet rich in phytoestrogens (i.e., “SOY”) reduces adiposity, adipose tissue inflammation, and IR in low aerobically fit female rats with and without ovarian function, yet whether the mechanism(s) for those benefits require E_2_ signaling remains unknown.

There is mounting evidence that a relationship exists between ERα expression and WAT function in both humans and rodents [10,11,12]. Young female knockout mice lacking ERα (αKO) have increased adiposity, impaired glucose tolerance [13], and increased WAT inflammation [14], whereas mice with functional ERα, but not ERβ (βKO), do not show this adverse phenotype [15]. This suggests that ERα, but not ERβ, is critical for providing protection against systemic metabolic and adipose dysfunction. Davis et al. [16] examined the specific effects of ERα in visceral WAT by selective deletion via viral knockdown of ERα to a single perigonadal fat pad in vivo and reported increased adipocyte size and WAT inflammation via increased proinflammatory cytokine gene expression compared to the untreated fat pad of the same animals. Those data in particular provide strong evidence that E_2_ via ERα in WAT is required for WAT immune and metabolic health. Recent data suggest that direct application of E_2_ on adipocytes (i.e., 3T3-L1 cells) induces translocation of ERα, but not ERβ, from the nucleus to the plasma membrane, which is associated with enhanced insulin stimulated phosphorylation of Akt and GLUT4 translocation [17]. Those effects were mimicked by PPT (4,4′,4″-(4-Propyl-[1*H*]-pyrazole-1,3,5-triyl)*tris*phenol), an ERα agonist, and were blocked by the ERα antagonist, MPP (1,3-*Bis*(4-hydroxyphenyl)-4-methyl-5-[4-(2-piperidinylethoxy)phenol]-1*H*-pyrazole dihydrochloride), suggesting that ERα signaling may directly augment insulin signaling in adipocytes [17].

E_2_ supplementation (i.e., hormone replacement therapy (HRT)) has been shown to attenuate weight gain and improve glucose tolerance in both postmenopausal women [18,19] and OVX rodents [20,21]; however, some clinical evidence suggests that HRT may increase cardiovascular risks (i.e., stroke) in susceptible postmenopausal women [22,23] and many women opt not to take it. Therefore, other therapeutic strategies are necessary to reduce the immune and metabolic disturbances seen in this population.

A soy-based diet rich in phytoestrogens is commonly used to help alleviate menopausal symptoms and improve overall metabolic health [24,25]. Soy intake has specifically been shown to reduce visceral adiposity, serum cytokines, cardiovascular, and metabolic risk factors in postmenopausal women [26,27] and in OVX rodents [28,29,30]. The main dietary phytoestrogens present in soy, genistein and daidzein, are aglycone metabolites with the ability to bind to and modulate both ERα and ERβ signaling [31]. Our previous findings demonstrated that SOY (i.e., an experimental rodent diet produced to mimic the nutritional profile provided by a human soy-based diet) fed ad libitum both reduced adiposity and improved metabolic parameters, including WAT inflammation, in OVX low-capacity running (LCR) rats [30].

The purpose of the current study was to determine if the molecular mechanism by which SOY improves individual components of metabolic health (e.g., body composition, adipose tissue health, insulin sensitivity, systemic metabolism) in females requires ER signaling. To this end, we tested the metabolic effects of SOY in older OVX female mice null for either ERα or ERβ. The results indicate that the systemic metabolic effects of SOY require signaling through ERα, a somewhat surprising finding in light of the available evidence that phytoestrogens are stronger ERβ over ERα agonists.

## 2. Materials and Methods

### 2.1. Study Design

**Figure 1:**
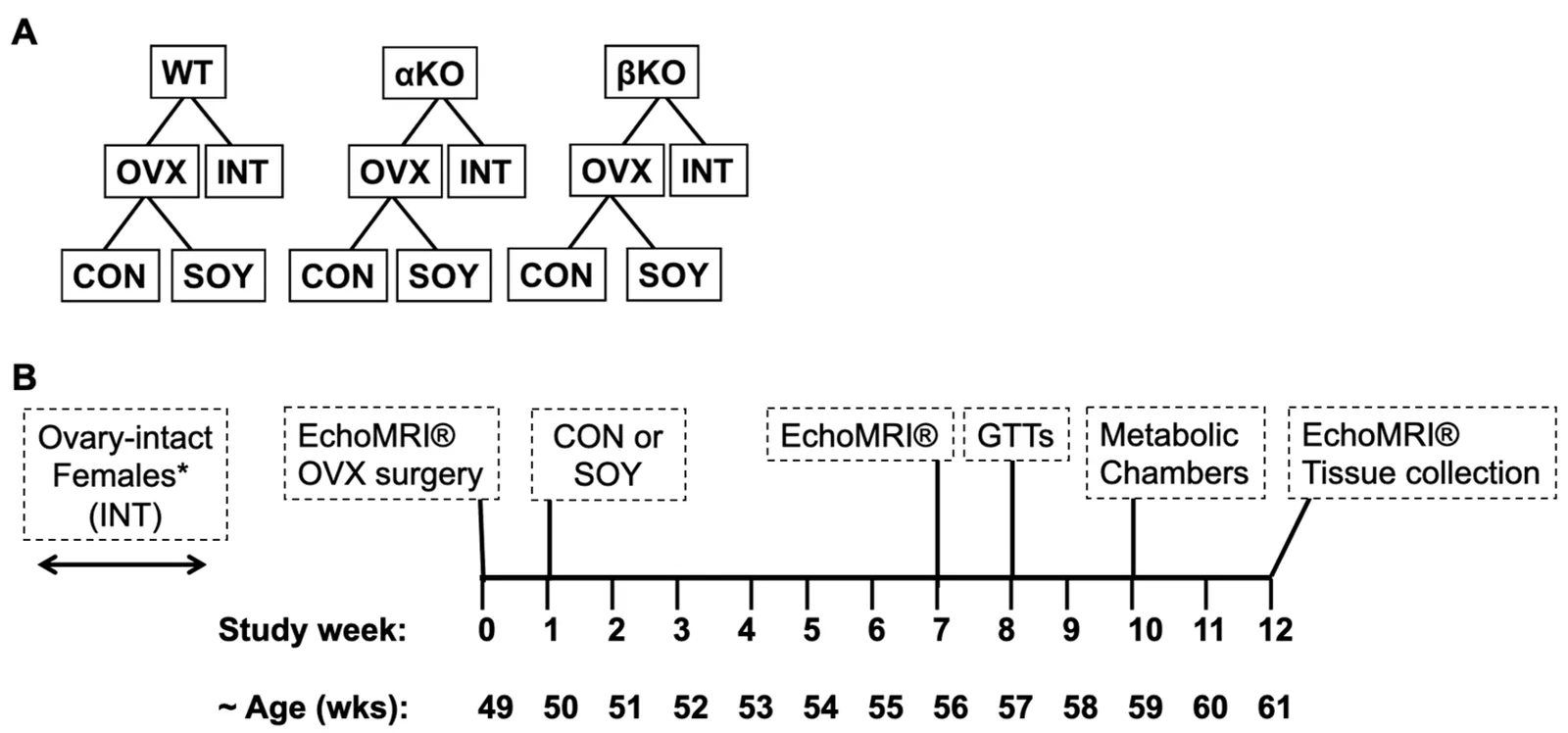
Study design and timeline. (**A**) Animal groupings: wildtype (WT), estrogen receptor alpha (αKO), and beta (βKO) female knockout mice either remained ovary-intact (INT) or were ovariectomized (OVX) and were introduced to either a phytoestrogen-rich (SOY) or a phytoestrogen-free (CON) diet one week post-OVX. (**B**) Timeline of study. * Ovary-intact mice were not assessed via EchoMRI^®^. GTT = glucose tolerance test.

Randomly cycling female (C57BL/6J background) αKO and βKO mice were pair-housed in a thermoneutral environment (26–28 °C) on a 12 h light/ 12 h dark cycle. ERα wildtype (WT) controls were used to validate replication of previous studies [16,32] and underwent all treatments similar to the αKO and βKO mice. All mice were initially provided with a low phytoestrogen content (i.e., “washout”) diet (150–250 mg/kg; #2018, Teklad Diets; Madison, WI, USA) and water ad libitum. One week post-OVX mice were randomized to receive one of two custom-made diets: a phytoestrogen-free (CON; <15 mg/kg phytoestrogens) or a phytoestrogen-rich diet (SOY, 585 mg/kg phytoestrogens [genistein and daidzein; aglycone equivalents]) purchased from Harlan Laboratories Inc. (Madison, WI, USA) (Figure 1). Regarding sample sizes across the 6 experimental groups, they ranged from 7 to 12/group. The discrepancies in *n* per group was because the mice were aged, which resulted in a small number dying prior to tissue collection (due to natural/age-related causes). Animals that died were not used for post-mortem analyses. In addition, likely due to the aging factor (i.e., increased surgery recovery risk), a few mice died within 48 h following OVX and thus could not be included. The histological analyses of adipose tissue sections included 4–5/group due to a limited number of high-quality images (e.g., free of technical images related to bad preps resulting in broken adipocytes and/or poor staining). For the FACS analyses on isolated adipocytes, some technological issues (e.g., insufficient cell yield) prevented more robust *n* in those experiments. For all other analyses, the *n*/group were as follows: αKO /CON *n* = 7; αKO /SOY *n* = 8; βKO CON *n* = 11; βKO /SOY *n* = 12; WT/CON *n* = 7; WT/SOY *n* = 8. The dietary details have been previously described [30], and phytoestrogen concentrations were validated in-house via HPLC. Body weight (BW) and food intake (FI) were monitored and recorded weekly. Ovary-intact WT, αKO, and βKO mice were examined as a separate cohort serving as a “pre-OVX” control group; these animals did not undergo OVX surgery, weekly FI, or body composition via EchoMRI^®^ measurements. Twelve weeks post-OVX, tissues were harvested from all animals for further analyses as described below. National Institutes of Health guidelines were strictly followed and all procedures were approved by the Institutional Animal Care and Use Committee at the University of Missouri prior to study initiation. αKO mice have been validated to not have functional ERα whereas βKO mice have been validated to exhibit functional ERα (i.e., ERα positive) yet lack genomic Erβ activity [33].

### 2.2. Ovariectomy (OVX) Surgeries

For OVX surgery, mice (~50 weeks old) were anesthetized and maintained under 2% isoflurane. A single 1 cm dorsal midline incision was made in the skin along the mid-lumbar region followed by two bilateral muscular incisions to expose the ovaries. OVX included removal of the whole ovary, ovarian bursa and part of the oviduct. After ovary removal, the skin incision was closed using skin sutures (4–0 Vicryl; Ethicon, Raritan, New Jersey, USA) and adhesive (3M VetBond Tissue Adhesive, Ethicon, Raritan, New Jersey, USA). Buprenorphine (Patterson Veterinary, Devens, MA, USA) was administered subcutaneously at a dose of 0.05 mg/kg immediately after surgery and post-operatively as needed. OVX effectiveness was determined at the conclusion of the study via verification of uterine atrophy.

### 2.3. Body Composition

Body fat and lean mass were measured by a non-invasive nuclear magnetic resonance imaging whole-body composition analyzer (EchoMRI^®^ 4in1/1100; Echo Medical Systems, Houston, TX, USA). EchoMRI^®^ was performed on conscious mice to gather pre-surgery data (week 0), mid-way through the study (week 7), and one day prior to sacrifice (12 weeks).

### 2.4. Glucose Tolerance Test (GTT) and Insulin Sensitivity

An intraperitoneal GTT was performed on ovary-intact mice (i.e., pre-OVX time point) and at 8 weeks post-OVX as previously described [5,34]. Briefly, following a six-hour fast, baseline blood (time 0) was collected from a nick in the tail vein and sampled by a hand-held glucometer (AlphaTRAK; Abott Laboratories, Abbott Park, IL, USA). Then, an intraperitoneal injection of a sterile solution of 50% dextrose (2 g/kg BW) was administered. Blood glucose measurements were taken at 15, 30, 45, 60, and 120 min following injection. Glucose area under the curve (AUC) above baseline was calculated. Homeostasis model assessment of IR (HOMA-IR) and adipocyte-IR (Adipo-IR) were used as surrogate measures of IR [35,36] and calculated using the following equations: HOMA-IR [fasting insulin (ng/mL) × fasting glucose (mg/dL)] and Adipo-IR [fasting insulin (ng/mL) × NEFA (mmol/L)].

### 2.5. Indirect Calorimetry and Physical Activity Assessment

Indirect calorimetry with a multidimensional beam break monitoring system (Promethion, Sable Systems International, Las Vegas, NV, USA) was used to assess total energy expenditure (TEE), resting energy expenditure (REE), and spontaneous physical activity (SPA) for ovary-intact and 10 weeks post-OVX mice. Manufacturer-constructed data analysis macros (Expedata Software v. 1.9.14, Sable Systems International) were used to gather averaged 12 h light or 12 h dark cycles. Animals were singly housed in the monitoring system and were allowed to acclimate to the environment 24 h prior to data collection. Cage SPA was calculated as the sum of the X, Y, and Z beam breaks. TEE and REE are presented as kcals/kg/12 h cycle or for 24 h, as indicated in the figures.

### 2.6. Fasting Circulating Markers

Blood was collected upon sacrifice via cardiac puncture and placed into 2.0 mL 7.5% EDTA-treated microcentrifuge tubes (Covidien Monoject, Cardinal Health, Dublin, OH, USA). The tubes were then centrifuged to obtain plasma, which was stored in the −80 °C freezer until further analysis. Commercial assays for fasting insulin, glucose, cholesterol, triglycerides (TG), low- and high-density lipoproteins (LDL, HDL) and nonesterified fatty acids (NEFA) were performed by a diagnostic laboratory (Comparative Clinical Pathology Services, Columbia, MO, USA) on an Olympus AU680 automated chemistry analyzer (Beckman-Coulter, Brea, CA, USA) according to the manufacturer’s guidelines. Plasma E_2_ was measured via radioimmunoassay at the Vanderbilt University Medical Center Hormone Assay Analytical Core (Vanderbilt University, Nashville, TN, USA) on KO mice only.

### 2.7. Histological Analyses

Formalin-fixed samples (*n* = 4–5/group) were processed through paraffin embedment, sectioned at 5 μm, and stained with hematoxylin and eosin for morphometric determinates, as previously described [37]. Sections were examined using an Olympus BX60 photomicroscope (Olympus, Melville, NY, USA) and photographed at 20× magnification with a Spot Insight digital camera (Diagnostic Instruments, Sterling Heights, MI, USA). An investigator, blinded to experimental conditions, collected four fields of view from each sample photograph. A representative photograph from one animal per group was selected. For cell size analysis, ~100 random adipocytes, representing at least four fields of view, were analyzed per animal using ImageJ software v. 1.52 (National Institutes of Health, public domain).

### 2.8. RNA Extraction and Real-Time PCR

Samples were homogenized in a Qiazol solution (Qiagen Catalog #79306) using a tissue homogenizer (TissueLyser LT, Qiagen, Valencia, CA, USA). Total RNA from combined retroperitoneal fat pads (i.e., visceral WAT) was isolated using the Qiagen’s Rneasy Lipid Tissue Kit and assayed using a Nanodrop spectrophotometer (Thermo Scientific, Wilmington, DE, USA) to assess purity and concentration. First-strand cDNA was synthesized from total RNA using the High Capacity cDNA Reverse Transcription kit (Applied Biosystems, Carlsbad, CA, USA). Quantitative real-time PCR was performed as previously described using the ABI StepOne Plus sequence detection system (Applied Biosystems) [38]. Primer sequences were designed using the NCBI Primer Design tool (Primers: IDT, Coralville, IA, USA) and provided in our prior publication [39]. A 20 μL reaction mixture containing 10 μL iTaq UniverSYBR Green SMX (BioRad, Hercules, CA, USA) and the appropriate concentrations of gene-specific primers plus 4 μL of cDNA template were loaded in each well of a 96-well plate. All PCR reactions were performed in duplicate. PCR was conducted with thermal conditions as follows: 95 °C for 10 min, followed by 40 cycles of 95 °C for 15 s and 60 °C for 45 s. A dissociation melt curve analysis was performed to verify the specificity of the PCR products. 18S primers were used to amplify the endogenous control product (i.e., HK gene). mRNA expression values are presented as 2^ΔCT^ whereby ΔCT = HK CT—gene of interest CT; data are expressed as fold difference relative to the respective control group.

### 2.9. Western Blot Analysis

Western blotting was performed as previously described [37,40]. Visceral WAT samples were pulverized and homogenized in Triton X-100 containing protease and phosphatase inhibitors using a tissue homogenizer (TissueLyser LT, Qiagen). WAT protein homogenates (5 μg protein/sample) and Laemmli buffer were then separated by SDS-PAGE, transferred to polyvinylidene difluoride membranes, and probed with primary antibodies (1:250–1:2000 in 5% bovine serum). Individual protein bands were quantified using a densitometer (Bio-Rad) and normalized to total protein. Antibodies purchased from Cell Signaling (Beverly, MA, USA) were GLUT4 (1:1000; #2213), phosphorylated Akt (pAkt) (1:250; #4060), and Akt (1:500; #4691). Purchased elsewhere were ERα (1:1000; #75635, Abcam, Waltham, MA, USA) and UCP1 (1:1000; #U6382, Sigma-Aldrich, St. Louis, MO, USA) antibodies.

### 2.10. Primary Cell Culture

#### 2.10.1. Isolation and Treatment of Cells

Primary stromal vascular cells (SVCs) including pre-adipocytes from C57BL/6 female WT mice were isolated and cultured in DMEM/F12 (1:1 invitrogen) containing 10% FBS, 1% Glutamax, and 0.1% Gentamicin, as previously described [41]. Adipocyte differentiation was induced (Day 0) in pre-adipocyte culture by treating confluent cells in medium containing 10% FBS, 0.5 mM isobutylmethylxanthine ([IBMX] Sigma-Aldrich; #15879), 1 μM dexamethasone (Sigma-Aldrich; #D4902), 5 μg/mL insulin (Sigma-Aldrich, #10516), and 1 μM rosiglitazone (Sigma-Aldrich; #R2408). Then, 48 h after induction, cells were maintained on maintenance medium containing 10% FBS and 5 μg/mL insulin. Maintenance medium was carefully replaced every two days until there were evident adipocytes (~Day 6), and then treatment occurred.

Adipocytes were pretreated for 48 h in dextran coated charcoal stripped 10% FBS and DMEM/F12 medium and incubated in vehicle/control conditions or 1,3-*Bis*(4-hydroxyphenyl)-4-methyl-5-[4-(2-piperidinylethoxy)phenol]-1*H*-pyrazoledihydrochloride (MPP; Tocris #1991), an ERα antagonist, or 4-[2-Phenyl-5,7-*bis* (trifluoromethyl) pyrazolo [1,5-*a*]pyrimidin-3-yl]phenol (PHTPP; Tocris #2662). A vehicle, E_2_ (1.0 μM, Sigma-Aldrich #E2758), or a phytoestrogen cocktail consisting of 25 μM genistein (Tocris #1110) and 25 μM daidzein (Tocris #1417) was added to the media for 24 h to pretreat. The phytoestrogen cocktail was used to mimic the dietary intervention in the mice. Finally, cells were treated with either vehicle or insulin (10 μg/mL) 30 min prior to collection.

#### 2.10.2. Collecting Cells

The media was immediately aspirated from each well following 30 min incubation with either vehicle or insulin treatment, and 50 μL of lysis buffer (Sigma, #R0278–50 mL) plus a protease and phosphatase inhibitor cocktail mix (Thermo-Fisher, Waltham, MA, USA #78440) was then immediately added to each well to gather lysates for protein analysis.

#### 2.10.3. Fluorescence Associated Cell Sorting (FACS)

Stromal vascular cells (SVCs) were isolated from perigonadal adipose tissue (a visceral depot) via collagenase digestion as previously described [4] with slight modifications and assessed for T cell and macrophage phenotype. The following fluorophore-conjugated antibodies were used: CD3ε-PerCP-Cy5.5 (BD Pharmingen, San Diego, CA, USA #561478), CD4a- PECy7 (BD Pharmingen, #561473), CD8a-Alexa Fluor 647 (BD Pharmingen, #561475), CD11c- BV51 and CD11b-BV711 (BD Pharmingen, # 563168, 562949). Gating strategies included dead cell discrimination and lymphocyte quantification based on forward/side scatter and included unstained cells, single stain, and FMO controls. Cells were immunophenotyped using a CyAN ADP Analyzer (Beckman Coulter, Inc., Indianapolis, IN, USA) and data were analyzed using Summit 5.2 (Beckman Coulter).

### 2.11. Statistics

Differences were determined using a 3 × 2 analysis of variance (ANOVA) to determine main effects of genotype (3 levels—WT, αKO, and βKO; G), main effects of diet (2 levels—CON vs. SOY; D), and genotype x diet interactions; significant interactions were followed to determine individual group differences via post hoc Tukey’s tests. Time course data were assessed using repeated measures 2 × 2 ANOVA (2 genotype levels as opposed to 3 due to WT mice not being used in these particular assessments). For cell culture studies, 3 replicates were performed, each using pooled adipocytes isolated from 4 WT mice; for cell culture experiments, groups were analyzed via independent samples *t*-tests. All data were analyzed using SPSS v24 (Chicago, IL, USA) and are presented as mean ± SEM, where *p* ≤ 0.05 was considered statistically significant. Power tests were performed based on our prior study [39].

## 3. Results

### 3.1. Role of ERα and ERβ on SOY-Mediated Effects on Body Composition Following OVX

**Figure 2:**
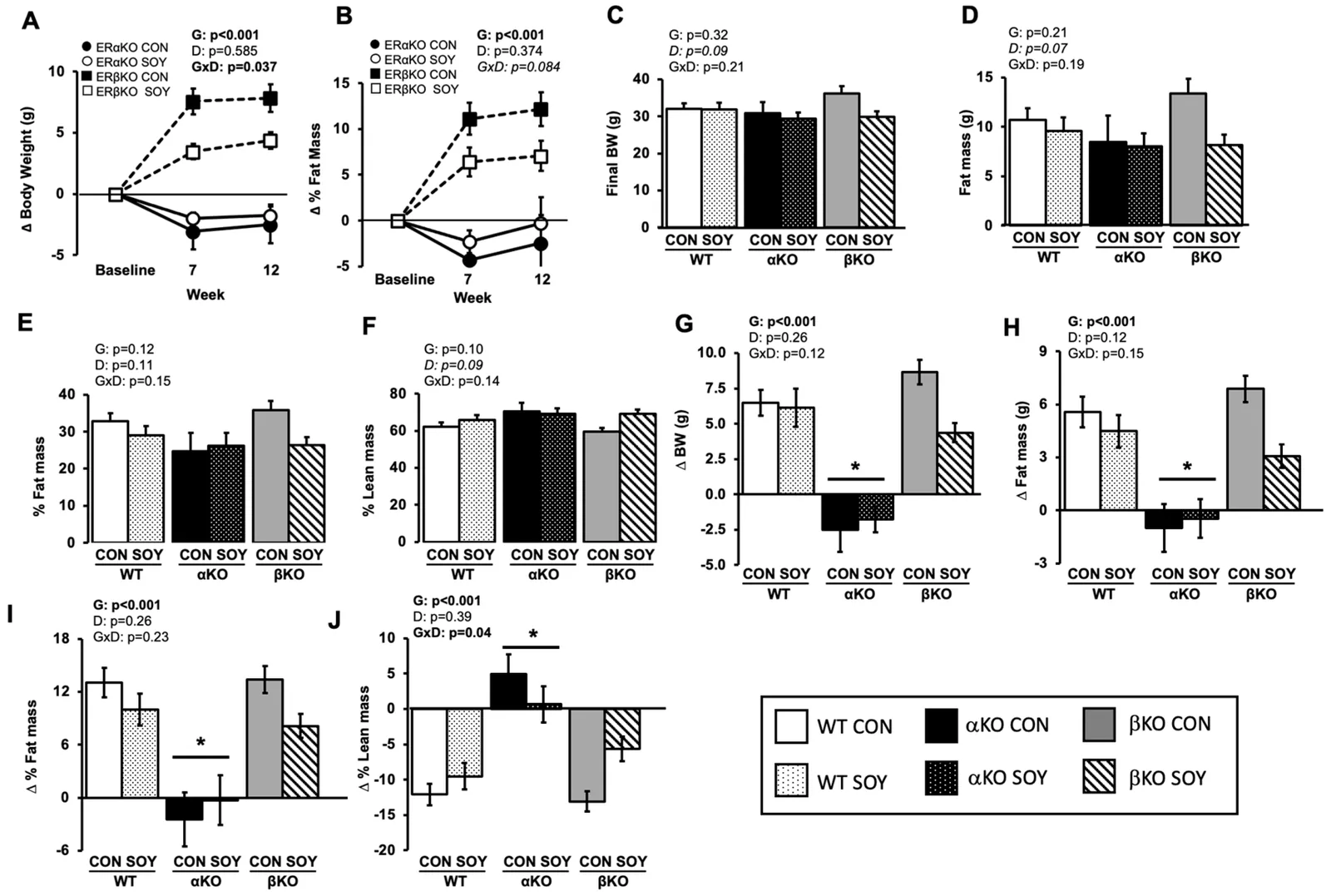
Effects of SOY on changes in body composition among genotypes. (**A**) Change in BW following OVX, (**B**) change in % fat mass following OVX, (**C**) final BW, (**D**) final fat mass, (**E**) final % fat mass, (**F**) final % lean mass, (**G**) total change in BW following OVX, (**H**) total change in fat mass following OVX, (**I**) total change in % fat mass, and (**J**) total change in % lean mass. Aged mice were OVX (at baseline) and fed ad libitum either a non-soy protein-based diet void of phytoestrogens (CON) or a soy protein-based diet rich in phytoestrogens (SOY). For A and B, 2-way ANOVAs with repeated measures were performed to determine main effects of genotype (G) and diet (D) as well as G × D interactions. For C-J, 2-way ANOVAs were performed using 3 genotype levels. Values are mean ± SEM; * Indicates significant difference (*p* < 0.05) from other genotypes by post hoc Tukey’s test.

As demonstrated in our prior study [39], OVX increased weight gain in βKO but not αKO mice, who tended to lose weight following OVX. Here, we demonstrate that SOY feeding attenuated weight gain, but only in βKO mice (Figure 2). SOY reduced final BW in the βKO mice by ~17%, which was not evident in the WT (0%) or αKO (6%) mice. This associated with a trend toward lowered fat mass (↓39%; *p* = 0.07) in the βKO mice, whereas smaller reductions in WT (↓10%) and αKO (↓5%) did not approach statistical significance. SOY also attenuated OVX-mediated reduction in lean mass in a genotype-specific manner such that ERα was required for that effect of SOY (Figure 2J; *p* = 0.04 G×D interaction).

**Figure 3:**
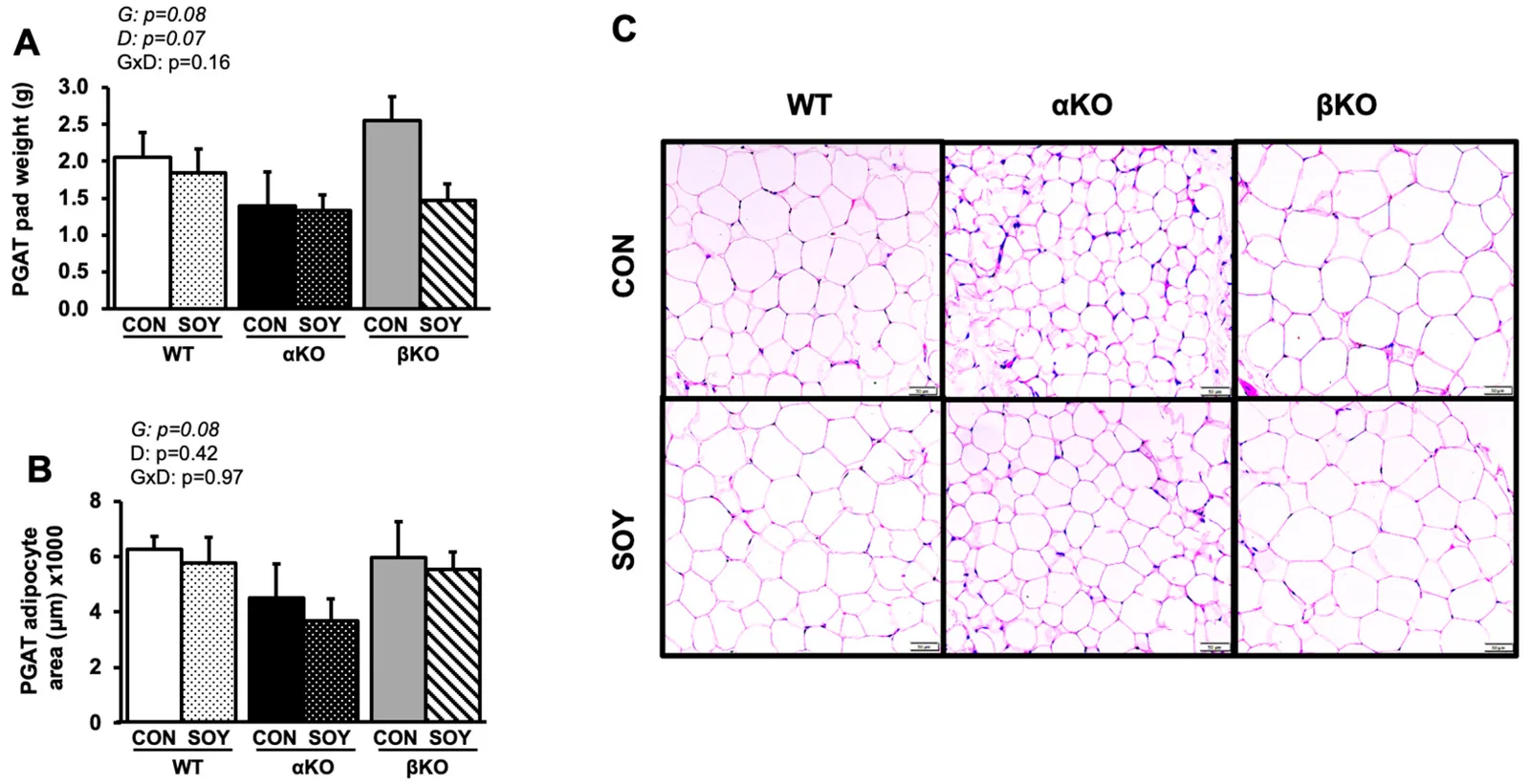
Effects of SOY on visceral adiposity and adipocyte phenotype following OVX. (**A**) Perigonadal (PGAT) fat pad weights (n = 7–12/group) and (**B**) adipocyte size (n = 4/group) with (**C**) representative histological images of white adipocytes for all genotypes. For A and B, 2-way ANOVA was used to determine main effects of genotype (G) and diet (D) as well as G × D interactions; values are presented as mean ± SEM.

As shown in Figure 3, SOY also tended to reduce perigonadal fat pad weight in a genotype-specific manner (Figure 3A, *p* = 0.07); differences between groups in perigonadal adipocyte size were not observed (Figure 3B,C). Within genotype, there was a 43% attenuation (*t* test: *p* < 0.01) of perigonadal pad weight with SOY in the βKO mice, whereas SOY hardly affected WT (11% reduction, NS) and had even less effect on αKO mice (4% reduction, NS).

### 3.2. SOY-Mediated Effects on Systemic Metabolism Following OVX

**Figure 4:**
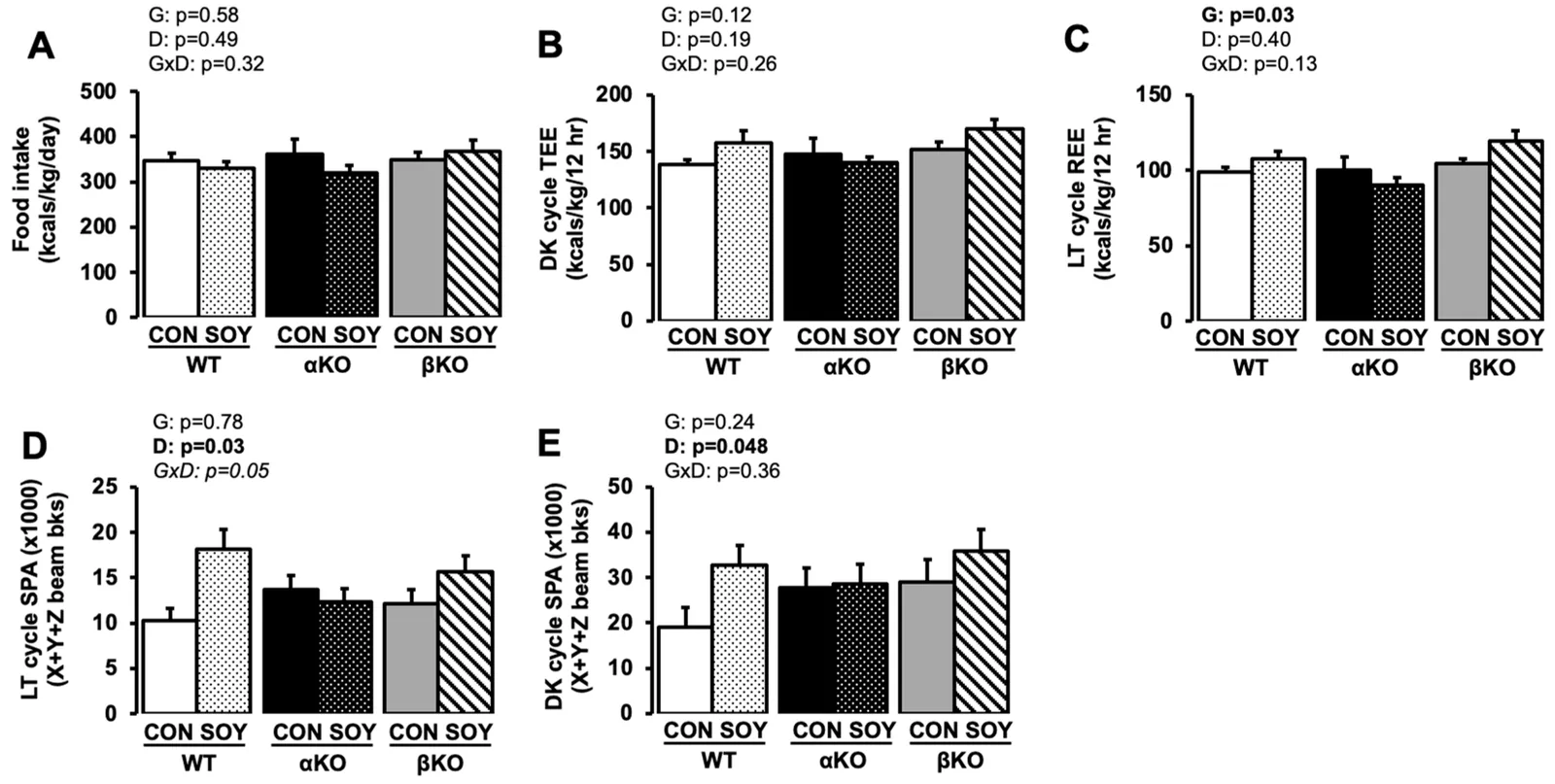
Effects of SOY on food intake and energy expenditure (EE) in βKO and αKO mice. (**A**) Food intake, (**B**) dark cycle (12 h) total EE (TEE), (**C**) light cycle (12 h) resting EE (REE) and (**D**) 12 h light and (**E**) dark cycle spontaneous physical activity (SPA). Two-way ANOVAs were performed to determine main effects of genotype (G) and diet (D) as well as G×D interactions (n = 7–12/group). Values are presented as mean ± SEM.

As shown in Figure 4, SOY did not significantly affect food intake (Figure 4A) or EE (Figure 4B,C). Surprisingly, SOY significantly increased cage locomotor activity (i.e., SPA), an effect not observed in αKO mice (Figure 4D, trend for G×D interaction).

### 3.3. SOY-Mediated Effects on Blood Lipids and Insulin Sensitivity Following OVX

Fasted serum lipids, glucose and insulin levels are presented in Table 1. Whereas OVX caused some degree of dyslipidemia in both genotypes, βKO mice were more adversely affected. However, SOY significantly lowered serum fasting cholesterol levels among αKO and βKO mice following OVX. Among βKO mice, SOY also improved LDL levels.

**Figure 5:**
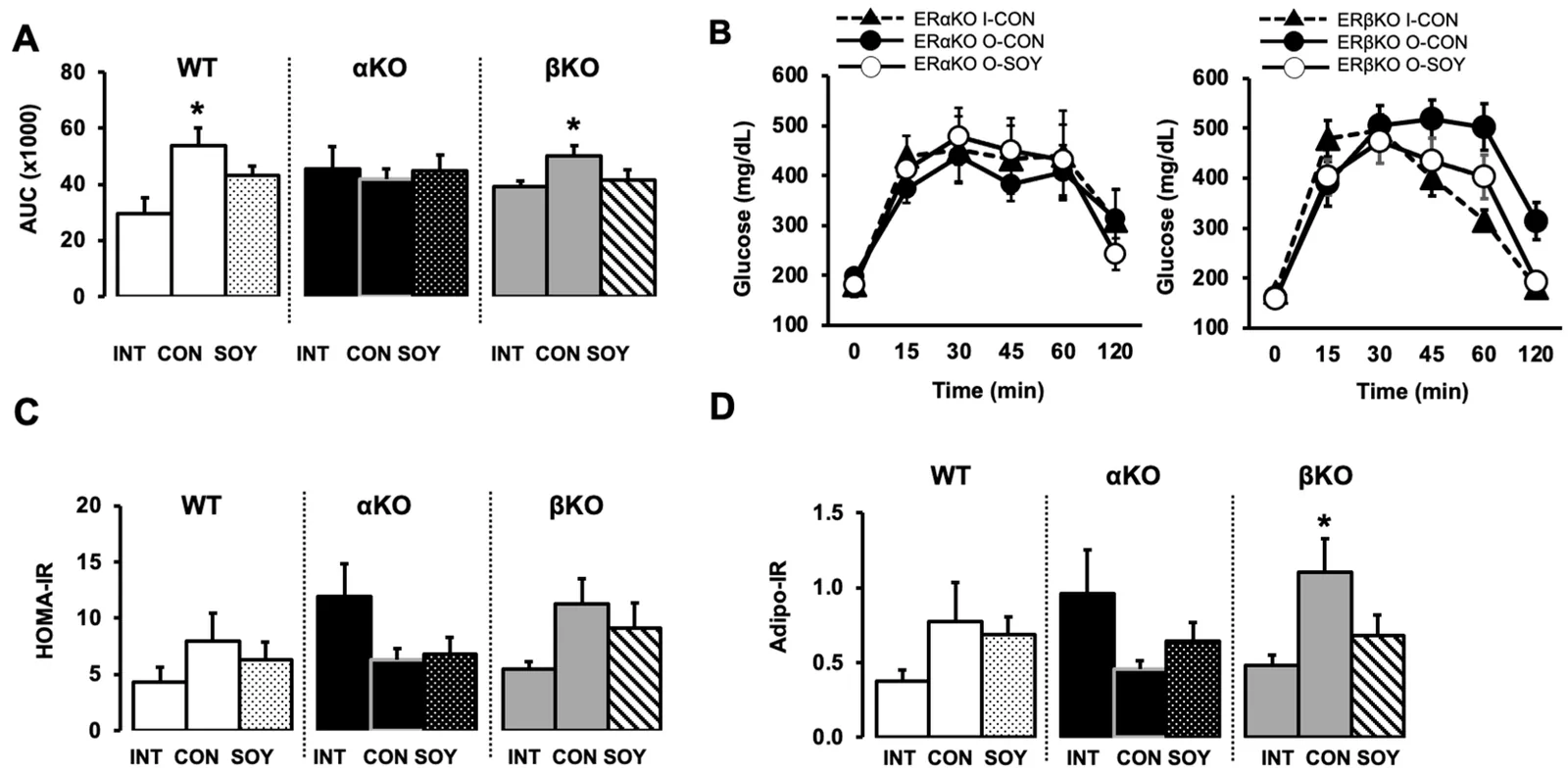
Effects of SOY on insulin sensitivity in OVX βKO and αKO mice. (**A**) Glucose tolerance test as glucose AUC, (**B**) GTT curves; (**C**) HOMA-IR and (**D**) Adipo-IR were used to assess insulin sensitivity in INT, and OVX on CON or SOY. One-way ANOVA was used to assess differences within genotypes, which were followed by post hoc Tukey’s tests. Values are presented as mean ± SEM (n = 7–12/group). (**A**) * *p* < 0.05 compared to INT within genotype; (**D**) * *p* < 0.05 compared to both other groups within genotype.

SOY improved glucose homeostasis following OVX in a genotype-specific manner (Figure 5). Improvements were only observed in WT and βKO genotypes, whereas αKO mice were unresponsive. SOY reduced glucose AUC by ~20% in WT and ~17% in βKO mice (Figure 5A,B), whereas there was no change in AUC for αKO mice fed SOY. SOY did not significantly affect HOMA-IR (Figure 5C), but within the βKO genotype, SOY attenuated the development of adipose tissue insulin resistance (Adipo-IR) by 39% (Figure 5D).

### 3.4. SOY-Mediated Effects on Adipose Tissue Markers of Insulin Sensitivity Following OVX

**Figure 6:**
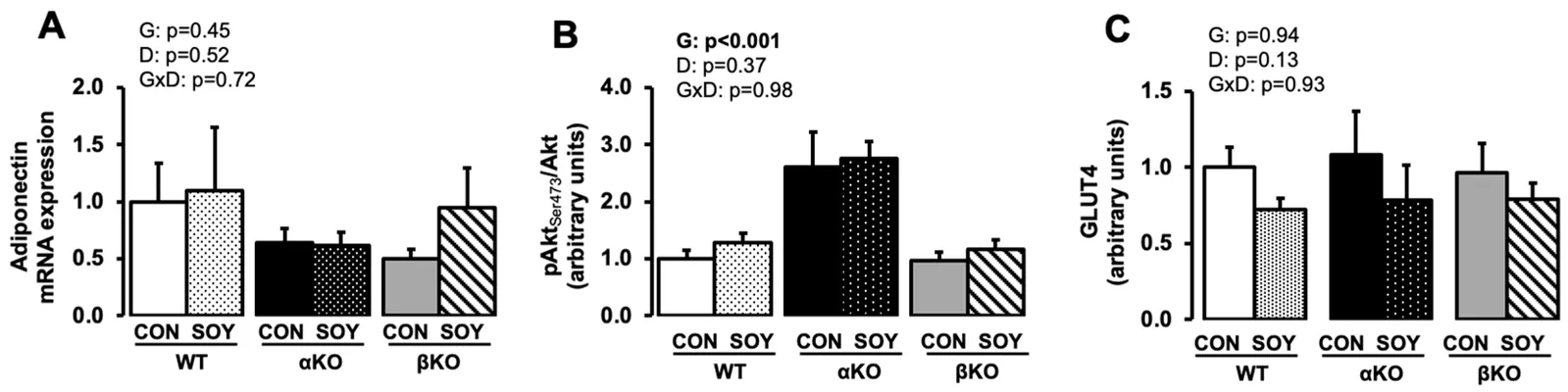
Effects of SOY on markers of WAT insulin sensitivity. (**A**) Adiponectin gene expression; (**B**) pAKT/AKT protein expression relative to OVX WT on CON group; and (**C**) GLUT4 protein expression relative to OVX WT CON. Two-way ANOVAs were performed to determine main effects of genotype (G) and diet (D) as well as GXD interactions. Values are presented as mean ± SEM (n = 7–12/group).

As shown in Figure 6, SOY did not significantly affect visceral WAT markers of insulin sensitivity. SOY-mediated increase in gene expression of adiponectin, the most robust protein indicator of adipocyte insulin sensitivity, was not statistically significant (Figure 6A). Basal level of insulin sensitivity, as determined by relative level of phosphorylated Akt under fasted conditions, was higher in the αKO mice compared to both WT and βKO mice (Figure 6B, *p* < 0.001 genotype main effect) but there were no significant effects of SOY. There were also no differences in GLUT4 protein in this basal/fasting condition.

### 3.5. SOY-Mediated Effects on Adipose Tissue Inflammation Following OVX

**Figure 7:**
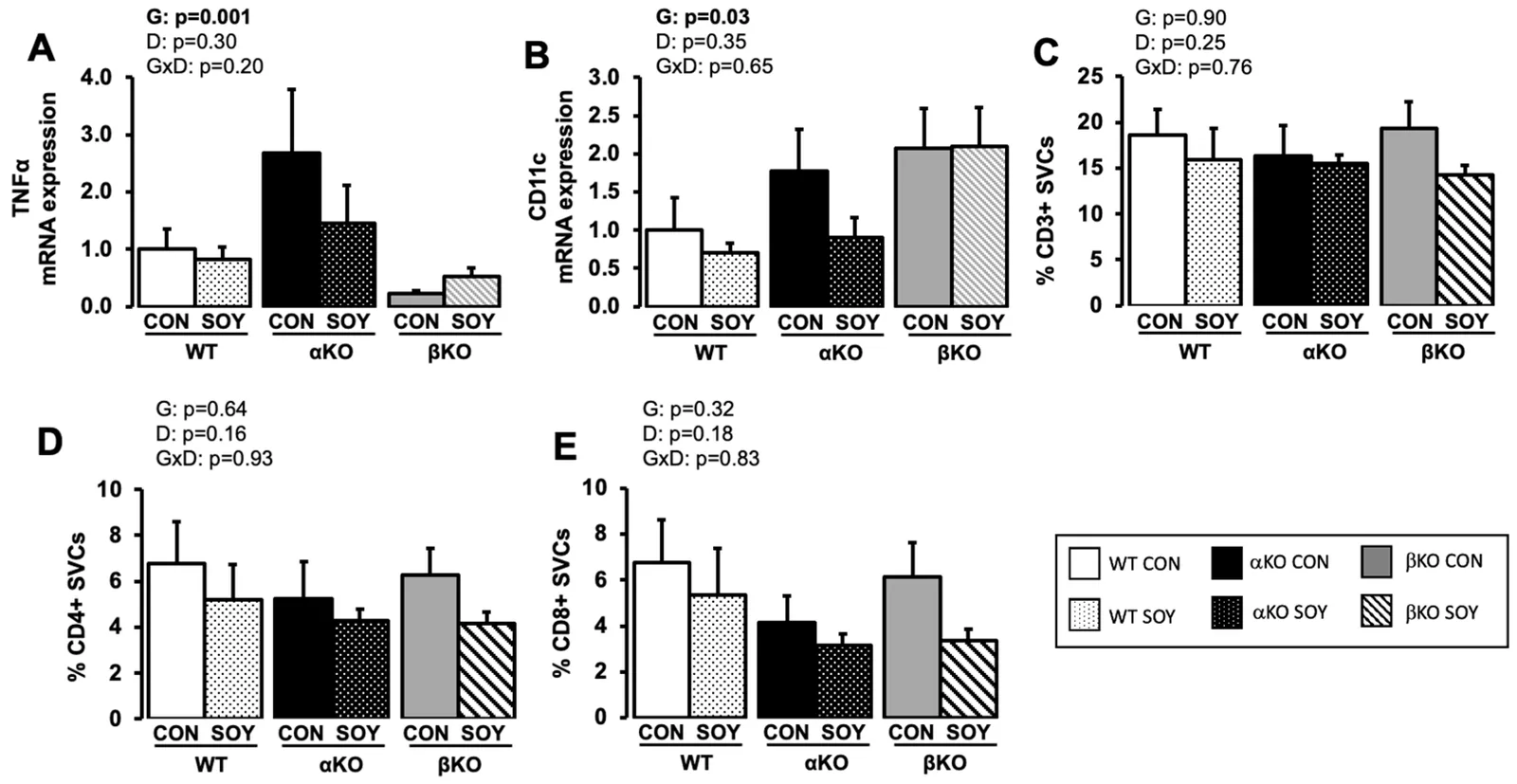
Effects of SOY on indicators of WAT inflammation. WAT gene expression examined for (**A**) TNFα and (**B**) CD11c. T lymphocyte accumulation of (**C**) %CD3+ SVCs, (**D**) %CD4+ SVCs, and I %CD8+ SVCs were examined in visceral, perigonadal, WAT via FACS. Two-way ANOVAs were performed to determine main effects of genotype (G) and diet (D) as well as G×D interactions. Values are presented as mean ± SEM. (**A**,**B**): n = 7–12/group; (**C**–**E**) (FACS): WT/CON n = 3; WT/SOY n = 8; aKO/CON n = 5; aKO/SOY n = 7; bKO/CON, n = 10; bKO/SOY, n = 11.

There were no differences between groups for any inflammatory makers that were measured in perigonadal adipose tissue. As shown in Figure 7, gene expression of the inflammatory cytokine, TNFα tended to be higher among αKO mice compared to the other genotypes (main effect of genotype; no significant individual group differences), yet was not affected by SOY (Figure 7A). The marker for inflammatory macrophages, CD11c, tended to be higher among βKO mice (main effect of genotype; yet no significant individual group differences, Figure 7B). We also performed preliminary assessments of immune cell (T-cell) infiltration using FACS and found no significant differences between groups (Figure 7C–E). It should be noted, however, that these experiments had relatively small ns and thus should be viewed as preliminary.

### 3.6. Effects of Phytoestrogen and Estradiol Exposure on Insulin Sensitivity in Primary Cultured Adipocytes

**Figure 8:**
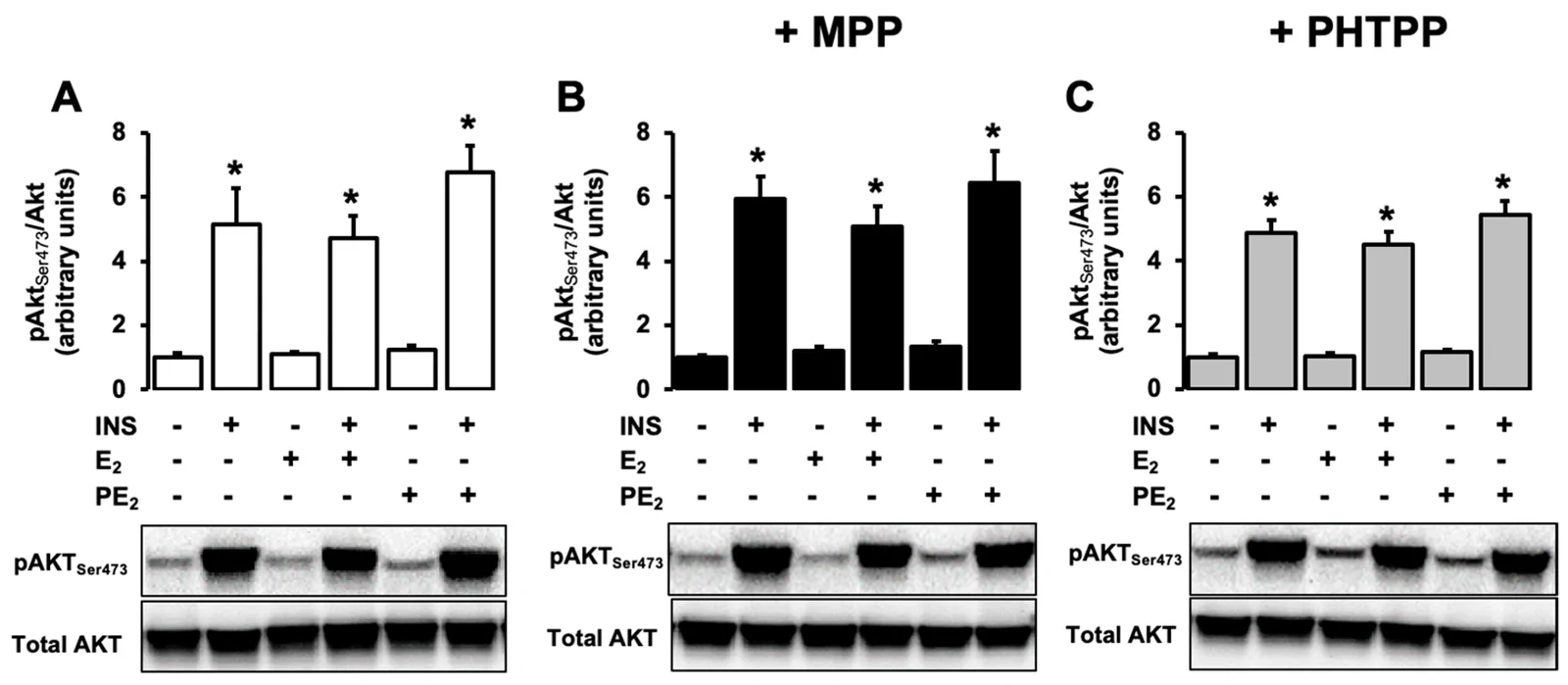
Effects of E_2_ and soy phytoestrogens on insulin-mediated phosphorylation of Akt in primary adipocyte cell culture. Insulin stimulation with (**A**) hormone only, (**B**) hormone + ERα antagonist (MPP), and (**C**) hormone + ERβ antagonist (PHTPP). Values are mean ± SEM, * *p* < 0.01 from non-insulin stimulated vehicle. INS = insulin, E_2_ = estrogen (1 µM), PE_2_ = phytoestrogen cocktail (genistein 25 µM + daidzein 25 µM), MPP = ERα antagonist (1 µM), and PHTPP = ERβ antagonist (1 µM).

**Figure 9:**
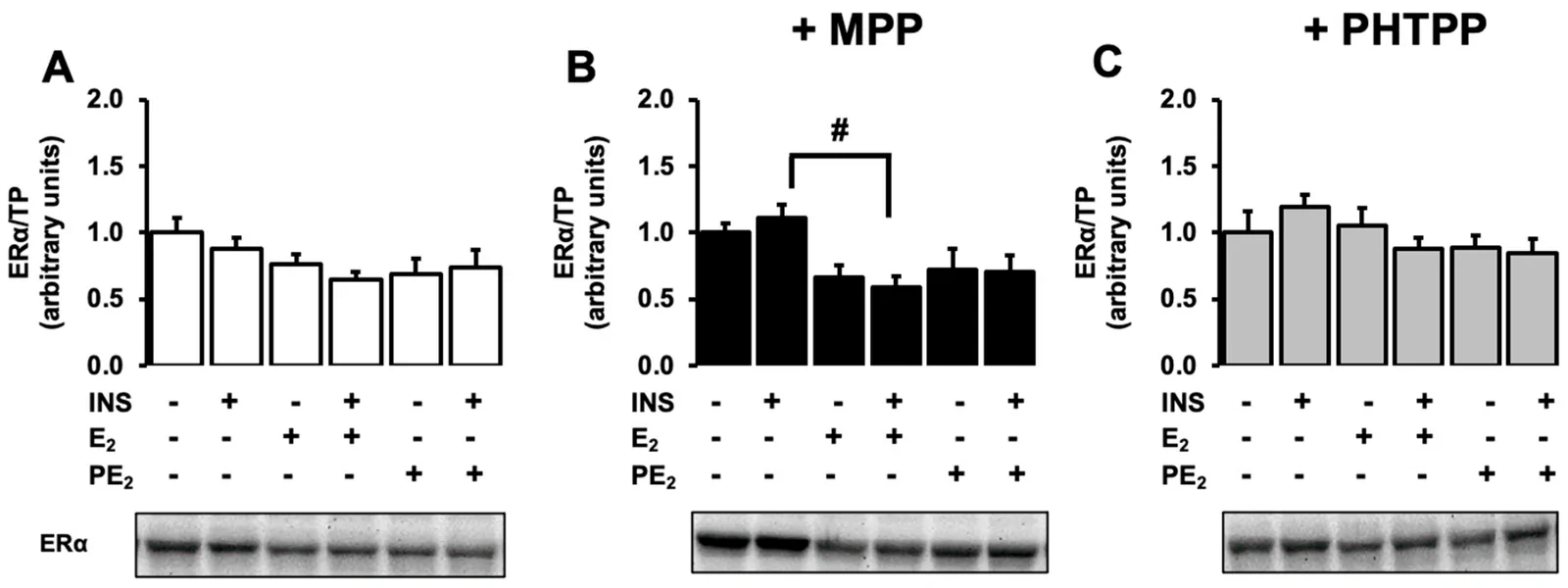
Individual and combined effects of insulin, E_2_, and phytoestrogens on ERα protein in cultured primary adipocytes. ERα protein relative to total protein (TP) in (**A**) hormone treatment only, (**B**) hormone + ERα antagonist (MPP), and (**C**) hormone + ERβ antagonist (PHTPP) in primary adipocytes. All One-way ANOVA within treatment group (i.e., no treatment, MPP, or PHTPP). Post hoc Tukey test was performed when differences were detected. Values are mean ± SEM, ^#^ *p* < 0.05 from insulin-stimulation with no hormone treatment. INS = insulin, E_2_ = estrogen (1 µM), PE_2_ = phytoestrogen cocktail (genistein 25 µM + daidzein 25 µM), MPP = ERα antagonist (1 µM), and PHTPP = ERβ antagonist (1 µM), and TP = total protein.

Since SOY improved glucose tolerance (Figure 5; *p* = 0.04) and Adipo-IR (*p* = 0.04) in the βKO mice, we examined the direct action of phytoestrogens present in SOY (i.e., by exposing primary cells to phytoestrogens as well as E_2_) on insulin-mediated pAkt/Akt in primary cultured adipocytes from WAT (Figure 8). As expected, insulin significantly increased pAkt protein, validating the cell culture model. However, the addition of E_2_ or a phytoestrogen cocktail did not alter this response, as indicated by protein expression of pAkt (Figure 8A). Blocking ERα via the ERα antagonist, MPP also did not affect insulin’s effect on pAkt protein expression (Figure 8B), nor did the presence of an ERβ antagonist (PHTPP) (Figure 8C). In all conditions, ERα protein was validated to be present (Figure 9). The only case in which ERα was altered was with joint exposure to insulin and E_2_ (lowering levels compared to cells exposed to MPP and insulin, Figure 9B).

## 4. Discussion

Weight gain and related metabolic dysfunction are important concerns for our aging population. While these affect both sexes, menopause in women poses unique metabolic risks due to the robust decline in estrogen, which is metabolically beneficial [42]. Meanwhile, plant-based diets have long been known to impact long-term health in many positive ways, including maintenance of a healthy body weight and overall cardiometabolic protection. Soy-based diets in particular have been met with skepticism due to the estrogenic properties of its phytoestrogens, but there do not appear to be any clinical or epidemiological literature to support harmful effects of soy or its phytoestrogen content. One caveat is that there are individuals who have allergic responses to soy; otherwise, the hesitation to consume soy-based products is likely not warranted. Soy phytoestrogens (e.g., genistein and daidzein) have been shown to reduce visceral adiposity, serum cytokines and cardiovascular and metabolic risk factors in postmenopausal women [26,27] and ovariectomized rodents [28,29,30]. Our previous study examined the effects of a soy-based diet rich in phytoestrogens (i.e., “SOY”) in female rats selectively bred for low running capacity (i.e., LCR) following ovariectomy (OVX) [30]. Ovariectomized LCR rats fed SOY exhibited reduced adiposity, improved insulin sensitivity and presented with better white adipose tissue (WAT) health (e.g., lower inflammatory expression of CD11c and IL-6) compared to OVX LCR rats fed the control corn-based diet (CON) [30]. We recently showed that SOY also improved bone strength in those rats [43]. Mechanisms are not known, but phytoestrogens have the ability to bind to and modulate estrogenic signaling through both ERα and ERβ, yet with weak affinity [24]. Thus, the purpose of the current study was to determine whether the molecular mechanisms for SOY-mediated improvements in metabolic and WAT health following OVX require ERα and/or ERβ.

ERs are known to affect insulin sensitivity. One clinical study showed the balance between ERα and ERβ protein in the subcutaneous WAT of menopausal women to be an important determinant for E_2_-mediated improvements in WAT insulin sensitivity, and that those women with a higher ERα /ERβ ratio had healthier WAT. In the present study, both wild-type (WT) (i.e., mice containing both ERα and ERβ) and βKO (i.e., mice positive for ERα but not ERβ) mice were used as controls to further assess the significance of ERα, with or without the presence of ERβ, and under the aging environment, which is more representative of human menopause; few studies have examined models of female hormone loss under aged conditions and none has investigated how such models may differentially respond to dietary phytoestrogens. We demonstrated the following: (1) Among older female OVX mice with normal ERα expression, SOY attenuated adiposity gain, improved insulin sensitivity, and reduced cholesterol levels; among mice null for ERα, SOY did not improve body composition or insulin sensitivity, yet did reduce cholesterol levels; (2) SOY did not affect ad libitum energy intake, but modestly increased locomotor activity, an effect not seen in ERα KO mice; (3) Despite these systemic improvements, SOY did not affect the indicators of WAT inflammation that were assessed here; and (4) Direct application of E_2_ or the soy phytoestrogens, daidzein and genistein on primary adipocytes did not increase insulin signaling as measured via insulin-mediated pAkt, and blockade of ERα using a chemical antagonist did not suppress insulin-induced pAkt.

We previously demonstrated that aged ovary-intact female αKO mice exhibit increased adiposity, insulin resistance (IR), and reduced energy expenditure (EE) compared to WT controls [39], similar to phenotypes seen in younger mice with ERα ablation from previous studies [13,16], confirming the important role ERα plays in systemic health. We also showed that OVX does not further exacerbate this negative phenotype in older ERα-null mice, and surprisingly, loss of E_2_ (via OVX) actually had a protective effect in this strain, exemplified by reduced visceral fat and adipocyte cell size and improved IR. Thus, it may not be surprising that SOY did not improve metabolic health among the αKO genotype, who started out “healthier” than the other OVX groups. In addition to αKO mice not being responsive to SOY, we found that the βKO mice tended to be the most responsive. Mechanisms for this enhanced responsiveness are unclear, but it is worth noting that adiponectin mRNA expression in the visceral WAT of the ovary-intact βKO mice was previously shown to be approximately three-fold higher compared to both (ovary-intact) WT and αKO mice, and the adiponectin-to-leptin ratio (indicative of adipocyte health) was approximately two-fold higher in βKO compared to αKO mice. In that previous study, OVX significantly increased Adipo-IR among βKO mice. Adiponectin is a potent anti-inflammatory and insulin-sensitizing adipokine secreted from the adipose tissue [44], and thus the adiponectin increase seen in the βKO mice following OVX may be a compensatory mechanism to protect these animals against developing metabolic disturbances. In support, prior studies in younger ERβ-null mice have shown them to exhibit improved insulin sensitivity despite weight gain, presumably related to their greater adiponectin synthesis [45]. Here, we show that SOY significantly improved Adipo-IR in the βKO mice but had no effect on this measure in the other groups. However, SOY did not affect WAT adiponectin levels in any group, nor did it affect basal pAKT levels in WAT, one measure of adipose tissue insulin sensitivity. Thus, the presence of ERβ may be protective against weight gain following OVX, while its absence may make it easier to gain weight albeit with preserved insulin sensitivity.

Despite phytoestrogens favoring ERβ, genomic signaling through this receptor does not appear to be necessary for metabolic benefits of SOY. One important caveat is that our βKO model is not a “complete” ERβ knockout, but one that lacks genomic signaling. Given that the vast majority of E_2_’s effects are genomic, this model has frequently been used since its development in the 1990s. However, we now know that ERs also mediate rapid/non-genomic signaling pathways [46,47,48], and thus effects of SOY demonstrated in WT and βKO mice may be, in part, mediated through non-genomic ERβ-mediated pathways.

In addition to improving glucose and lipid metabolism, SOY was also protective against OVX-mediated reductions in SPA, a major contributor to the weight gain following menopause in humans and OVX in rodents. Since this only occurred in WT and βKO mice, this finding is suggestive that ERα is responsible for SOY’s ability to increase SPA. This was also associated with preservation of lean mass in those mice with functional ERα. Although the idea that a dietary compound has the ability to augment physical activity behavior is unusual, isoflavone metabolites from SOY are able to cross the blood–brain barrier [49], and have been shown to affect ERα-selective ventromedial hypothalamic neurons that promote locomotion [50].

AT inflammation is linked with IR [51]. Visceral AT-specific ERα knockdown in mice results in reduced glucose clearance in the affected tissue indicating metabolic compromise, as well as increased inflammation [16]. This suggests that the IR induced by suppression of ERα may be due to increased WAT inflammation. However, there were no detectable differences in inflammatory variables between genotypes in our previous study and SOY also did not affect inflammatory gene expression or immune cell infiltration in this older OVX model in the present study. The lack of differences in inflammatory expression may have been due to the fact that obesity was not induced (e.g., via a high-fat fed) which is known to exaggerate an inflammatory response. Future studies should use a high-fat diet combined with SOY to determine whether SOY has anti-inflammatory effects in that setting.

The major isoflavones that have contributed to favorable health outcomes include genistein and daidzein, although these are not the only isoflavones present in soy [24]. These isoflavones have been studied as separate isolates to examine their individual effects in rodents. Supplementation of 0.1% genistein per day for one month in post-OVX Sprague Dawley rats improved fatty acid oxidation in visceral WAT, reduced adipocyte size and serum pro-inflammatory markers (leptin and TNFα), and improved insulin sensitivity, measured via HOMA-IR [52]. Diet-induced obese male mice with IR were placed on daidzein (1.0 g/kg of chow) for three months, which reduced visceral WAT adipocyte size and pro-inflammatory gene expression (i.e., TNFα) and macrophage expression (CD11c, F4/80, and monocyte chemoattractant protein 1 (MCP1)) with increased adiponectin gene expression [53]. There were also improvements in glucose tolerance compared to mice without daidzein supplementation [53]. Further investigation suggested the effects of daidzein was also evident in adipocytes alone (i.e., 3T3-L1 adipocytes), possibly contributing to the systemic effects seen in mice [53,54]. A 2025 study assessed the effects of E_2_ [10 nM], as well as phytoestrogens (daidzein [10–150 nM] and genistein [10 nM–50 uM]), on GLUT4 expression in 3T3-L1 adipocytes. They showed that both daidzein and genistein (as well as E_2_ [10 nM]) at a low dose did in fact increase GLUT4 expression, and this was abrogated by an ESR1 antagonist, MPP, the same one used in the present study [55]. However, this was dose-dependent, as at higher doses, the phytoestrogens reduced GLUT4 at mRNA and protein levels, effects that were also dependent upon ESR1. We did not assess GLUT4, but did not find differences in pAkt (which is upstream of GLUT4 translocation). Importantly, in comparison to the former study by Gregorio et al., ours used genestein and daidzein concentrations on the higher end [25 uM] and an E_2_ dose that was much higher [1 uM], which also did not produce an effect. Thus, our high doses may have masked effects we would have seen had we used lower concentrations and thus should not be used to rule out the hypothesis that phytoestrogens improve IR via their activation of ESR1 in adipose tissue. We also did not examine GLUT4 translocation, which has been shown to be highly affected by ERα in 3T3-L1 cells [17]. Additional cell culture studies are necessary to more precisely interrogate how phytoestrogens affect insulin signaling pathways in adipocytes—such studies should perform dose response curves and more accurately measure insulin sensitivity using functional in vitro assays.

Strengths and limitations: The use of older (>1 year) female αKO and βKO mice provided an additional aging stress, which has not been previously investigated in these knockout models. This approach is more representative of age-related hormonal changes typically observed in women. Another strength is the nutritional matching of the SOY and CON diets, which differed only in protein source (soy vs. corn-based) and phytoestrogen content (validated to be void in the CON diet). Finally, we collected a comprehensive portfolio of tissue-specific, systemic/in vivo, and in vitro data to assess the effects of ad libitum SOY intake on physiological metabolism as well as feeding and locomotor behavior. However, the study was limited by the following: (1) an imperfect ERβ knockout model, which displays lack of ERβ DNA binding, but not non-genomic signaling pathways; (2) insufficient data to make definitive conclusions on WAT inflammatory populations; (3) lack of head-to-head comparison to young mice, and/or age-matched male mice, to truly assess and differentiate effects of sex and aging; and (4) lack of dose response curves and in vitro functional assays. Future studies should address these issues. In addition, we used commercially designed diets to contain elevated levels of the major phytoestrogens, genistein and daidzein. While the concentrations of these phytoestrogens were validated in the diets, we did not assess plasma levels. In addition, it should be noted that soy protein contains numerous other isoflavones that also may have played a role in the outcomes observed, whereas the diets were enriched specifically in the phytoestrogens highlighted here. Finally, it is critically important to take this study’s findings within proper context. That is, these data were all generated in rodent models and cannot be directly applied to the human population until they are validated by clinical trials, which are urgently needed.

## 5. Conclusions

Due to the increasing propensity for obesity-related pathologies in menopausal women, and increased demand for ‘natural’ therapeutic dietary strategies to reduce these disease risks, these novel findings add to the growing body of literature showing therapeutic effects of a soy-based diet, particularly regarding insulin sensitivity. Further, these findings reveal that the mechanism by which SOY improves systemic metabolism and insulin sensitivity following OVX does not require genomic signaling through ERβ, the receptor most often associated with phytoestrogen effects due to evidence that phytoestrogens bind ERβ more readily than ERα. Further, the data indicate that older αKO mice are, in addition to being unresponsive to the negative metabolic effects of OVX, also immune to most of the positive metabolic effects of dietary SOY, whereas older βKO mice experienced increases in adiposity and insulin resistance following OVX that were, in some cases, improved by SOY. Future studies should address mechanisms, including brain-mediated mechanisms that may help explain SOY’s protective effects on systemic metabolism and behavior, as well as perform these studies on aging male mice.

## Figures and Tables

**Table 1:** Fasting blood profiles.

	αKO Mice			βKO Mice		
	I-CON	O-CON	O-SOY	I-CON	O-CON	O-SOY
Insulin (ng/mL)	1.29 ± 0.25	0.77 ± 0.18	0.78 ± 0.12	0.68 ± 0.08 ^c^	1.32 ± 0.24 ^a^	0.80 ± 0.16
Glucose (mg/dL)	219.3 ± 31.8	242.9 ± 16.8	187.6 ± 26.0	208.5 ± 14.2	219.2 ± 15.3	239.3 ± 15.1
Cholesterol (mg/dL)	68.7 ± 10.8	80.0 ± 6.1	52.4 ± 11.1 ^b^	74.3 ± 3.5	87.6 ± 5.2	69.2 ± 5.3 ^b^
LDL (mg/dL)	5.14 ± 0.59	7.14 ± 1.06	5.00 ± 0.65	4.80 ± 0.20	9.00 ± 0.74 ^a^	6.67 ± 0.78 ^a,b^
HDL (mg/dL)	32.1 ± 5.4	41.3 ± 2.2	29.1 ± 6.8 ^a^	34.9 ± 1.3 ^a^	39.7 ± 2.0	36.2 ± 1.7
Triglycerides (mg/dL)	133.57 ± 10.0	149.3 ± 5.9	144.9 ± 5.8	140.0 ± 5.5	147.0 ± 8.5	147.8 ± 5.5
NEFA (mmol/L)	0.70 ± 0.12	0.77 ± 0.07	0.81 ± 0.11	0.74 ± 0.07	0.88 ± 0.07	0.88 ± 0.08

Effects of ovariectomy (OVX) and SOY on fasting blood profiles. Values presented as mean ± SEM; n = 7–12/group. I = intact control; O = OVX; ^a^ *p* < 0.05 compared to intact-control within genotype; ^b^ *p* < 0.05 compared to OVX-control within genotype; ^c^ *p* < 0.05 αKO compared to βKO intact-control.

## Data Availability

All raw data are available upon request.

## Abbreviations

The following abbreviations are used in this manuscript:

Adipo-IR	adipose tissue insulin resistance
CON	low phytoestrogen diet
E_2_	17β-estradiol
EE	energy expenditure
ERa/ERα	estrogen receptor alpha
ERb/ERβ	estrogen receptor beta;
HOMA-IR	homeostatic model assessment of insulin resistance
INT	intact control
IR	insulin resistance
MPP	ERα antagonist
OVX	ovariectomized
PGAT	perigonadal adipose tissue
PHTPP	ERβ antagonist
PPT	ERα agonist
REE	resting energy expenditure
SOY	high phytoestrogen diet
SPA	spontaneous physical activity
TEE	total energy expenditure
WAT	white adipose tissue
WT	wildtype
αKO	estrogen receptor alpha knockout
βKO	estrogen receptor beta knockout

## References

[1] Bluher M. (2013). Adipose tissue dysfunction contributes to obesity related metabolic diseases. Best Pract. Res. Clin. Endocrinol. Metab..

[2] Bluher M. (2009). Adipose tissue dysfunction in obesity. Exp. Clin. Endocrinol. Diabetes.

[3] Lovejoy J.C., Champagne C.M., de Jonge L., Xie H., Smith S.R. (2008). Increased visceral fat and decreased energy expenditure during the menopausal transition. Int. J. Obes..

[4] Vieira Potter V.J., Strissel K.J., Xie C., Chang E., Bennett G., Defuria J., Obin M.S., Greenberg A.S. (2012). Adipose tissue inflammation and reduced insulin sensitivity in ovariectomized mice occurs in the absence of increased adiposity. Endocrinology.

[5] Vieira-Potter V.J., Padilla J., Park Y.M., Welly R.J., Scroggins R.J., Britton S.L., Koch L.G., Jenkins N.T., Crissey J.M., Zidon T. (2015). Female rats selectively bred for high intrinsic aerobic fitness are protected from ovariectomy-associated metabolic dysfunction. Am. J. Physiol. Regul. Integr. Comp. Physiol..

[6] Razmjou S., Bastard J.P., Doucet E., Rabasa-Lhoret R., Fellahi S., Lavoie J.M., Prud’homme D. (2016). Effect of the menopausal transition and physical activity energy expenditure on inflammatory markers: A MONET group study. Menopause.

[7] Stefanska A., Bergmann K., Sypniewska G. (2015). Metabolic Syndrome and Menopause: Pathophysiology, Clinical and Diagnostic Significance. Adv. Clin. Chem..

[8] Barros R.P., Gustafsson J.A. (2011). Estrogen receptors and the metabolic network. Cell Metab..

[9] Nilsson S., Makela S., Treuter E., Tujague M., Thomsen J., Andersson G., Enmark E., Pettersson K., Warner M., Gustafsson J.A. (2001). Mechanisms of estrogen action. Physiol. Rev..

[10] Nilsson M., Dahlman I., Ryden M., Nordstrom E.A., Gustafsson J.A., Arner P., Dahlman-Wright K. (2007). Oestrogen receptor alpha gene expression levels are reduced in obese compared to normal weight females. Int. J. Obes..

[11] Lizcano F., Guzman G. (2014). Estrogen deficiency and the origin of obesity during menopause. Biomed. Res. Int..

[12] Monteiro R., Teixeira D., Calhau C. (2014). Estrogen signaling in metabolic inflammation. Mediat. Inflamm..

[13] Heine P.A., Taylor J.A., Iwamoto G.A., Lubahn D.B., Cooke P.S. (2000). Increased adipose tissue in male and female estrogen receptor-alpha knockout mice. Proc. Natl. Acad. Sci. USA.

[14] Ribas V., Nguyen M.T., Henstridge D.C., Nguyen A.K., Beaven S.W., Watt M.J., Hevener A.L. (2010). Impaired oxidative metabolism and inflammation are associated with insulin resistance in ERalpha-deficient mice. Am. J. Physiol. Endocrinol. Metab..

[15] Ohlsson C., Hellberg N., Parini P., Vidal O., Bohlooly Y.M., Rudling M., Lindberg M.K., Warner M., Angelin B., Gustafsson J.A. (2000). Obesity and disturbed lipoprotein profile in estrogen receptor-alpha-deficient male mice. Biochem. Biophys. Res. Commun..

[16] Davis K.E., Neinast M.D., Sun K., Skiles W.M., Bills J.D., Zehr J.A., Zeve D., Hahner L.D., Cox D.W., Gent L.M. (2013). The sexually dimorphic role of adipose and adipocyte estrogen receptors in modulating adipose tissue expansion, inflammation, and fibrosis. Mol. Metab..

[17] Campello R.S., Fatima L.A., Barreto-Andrade J.N., Lucas T.F., Mori R.C., Porto C.S., Machado U.F. (2017). Estradiol-induced regulation of GLUT4 in 3T3-L1 cells: Involvement of ESR1 and AKT activation. J. Mol. Endocrinol..

[18] Musatov S., Chen W., Pfaff D.W., Mobbs C.V., Yang X.J., Clegg D.J., Kaplitt M.G., Ogawa S. (2007). Silencing of estrogen receptor alpha in the ventromedial nucleus of hypothalamus leads to metabolic syndrome. Proc. Natl. Acad. Sci. USA.

[19] Ryan A.S., Nicklas B.J., Berman D.M. (2002). Hormone replacement therapy, insulin sensitivity, and abdominal obesity in postmenopausal women. Diabetes Care.

[20] Xu Y., Nedungadi T.P., Zhu L., Sobhani N., Irani B.G., Davis K.E., Zhang X., Zou F., Gent L.M., Hahner L.D. (2011). Distinct hypothalamic neurons mediate estrogenic effects on energy homeostasis and reproduction. Cell Metab..

[21] Stubbins R.E., Holcomb V.B., Hong J., Nunez N.P. (2012). Estrogen modulates abdominal adiposity and protects female mice from obesity and impaired glucose tolerance. Eur. J. Nutr..

[22] Nelson H.D., Walker M., Zakher B., Mitchell J. (2012). Menopausal hormone therapy for the primary prevention of chronic conditions: A systematic review to update the U.S. Preventive Services Task Force recommendations. Ann. Intern. Med..

[23] Sood R., Faubion S.S., Kuhle C.L., Thielen J.M., Shuster L.T. (2014). Prescribing menopausal hormone therapy: An evidence-based approach. Int. J. Womens Health.

[24] Bhathena S.J., Velasquez M.T. (2002). Beneficial role of dietary phytoestrogens in obesity and diabetes. Am. J. Clin. Nutr..

[25] Cederroth C.R., Vinciguerra M., Gjinovci A., Kuhne F., Klein M., Cederroth M., Caille D., Suter M., Neumann D., James R.W. (2008). Dietary phytoestrogens activate AMP-activated protein kinase with improvement in lipid and glucose metabolism. Diabetes.

[26] de Kleijn M.J., van der Schouw Y.T., Wilson P.W., Grobbee D.E., Jacques P.F. (2002). Dietary intake of phytoestrogens is associated with a favorable metabolic cardiovascular risk profile in postmenopausal U.S. women: The Framingham study. J. Nutr..

[27] Panneerselvam S., Packirisamy R., Bobby Z., Sridhar M.G. (2016). Protective effect of soy isoflavones (from glycine max) on adipose tissue oxidative stress and inflammatory response in an experimental model of post-menopausal obesity: The molecular mechanisms. Biochem. Anal. Biochem..

[28] Sankar P., Zachariah B., Vickneshwaran V., Jacob S.E., Sridhar M.G. (2015). Amelioration of oxidative stress and insulin resistance by soy isoflavones (from Glycine max) in ovariectomized Wistar rats fed with high fat diet: The molecular mechanisms. Exp. Gerontol..

[29] Russell A.L., Grimes J.M., Cruthirds D.F., Westerfield J., Wooten L., Keil M., Weiser M.J., Landauer M.R., Handa R.J., Wu T.J. (2017). Dietary isoflavone-dependent and estradiol replacement effects on body weight in the ovariectomized (OVX) rat. Horm. Metab. Res..

[30] Cross T.L., Zidon T.M., Welly R.J., Park Y.M., Britton S.L., Koch L.G., Rottinghaus G.E., de Godoy M.R.C., Padilla J., Swanson K.S. (2017). Soy Improves Cardiometabolic Health and Cecal Microbiota in Female Low-Fit Rats. Sci. Rep..

[31] Oseni T., Patel R., Pyle J., Jordan V.C. (2008). Selective estrogen receptor modulators and phytoestrogens. Planta Med..

[32] Naaz A., Zakroczymski M., Heine P., Taylor J., Saunders P., Lubahn D., Cooke P.S. (2002). Effect of ovariectomy on adipose tissue of mice in the absence of estrogen receptor alpha (ERalpha): A potential role for estrogen receptor beta (ERbeta). Horm. Metab. Res..

[33] Lubahn D.B., Moyer J.S., Golding T.S., Couse J.F., Korach K.S., Smithies O. (1993). Alteration of reproductive function but not prenatal sexual development after insertional disruption of the mouse estrogen receptor gene. Proc. Natl. Acad. Sci. USA.

[34] Wainright K.S., Fleming N.J., Rowles J.L., Welly R.J., Zidon T.M., Park Y.M., Gaines T.L., Scroggins R.J., Anderson-Baucum E.K., Hasty A.H. (2015). Retention of sedentary obese visceral white adipose tissue phenotype with intermittent physical activity despite reduced adiposity. Am. J. Physiol. Regul. Integr. Comp. Physiol..

[35] Matthews D.R., Hosker J.P., Rudenski A.S., Naylor B.A., Treacher D.F., Turner R.C. (1985). Homeostasis model assessment: Insulin resistance and beta-cell function from fasting plasma glucose and insulin concentrations in man. Diabetologia.

[36] Sondergaard E., Espinosa De Ycaza A.E., Morgan-Bathke M., Jensen M.D. (2017). How to Measure Adipose Tissue Insulin Sensitivity?. J. Clin. Endocrinol. Metab..

[37] Thyfault J.P., Rector R.S., Uptergrove G.M., Borengasser S.J., Morris E.M., Wei Y., Laye M.J., Burant C.F., Qi N.R., Ridenhour S.E. (2009). Rats selectively bred for low aerobic capacity have reduced hepatic mitochondrial oxidative capacity and susceptibility to hepatic steatosis and injury. J. Physiol..

[38] Padilla J., Jenkins N.T., Vieira-Potter V.J., Laughlin M.H. (2013). Divergent phenotype of rat thoracic and abdominal perivascular adipose tissues. Am. J. Physiol. Regul. Integr. Comp. Physiol..

[39] Zidon T.M., Padilla J., Fritsche K.L., Welly R.J., McCabe L.T., Stricklin O.E., Frank A., Park Y., Clegg D.J., Lubahn D.B. (2020). Effects of ERbeta and ERalpha on OVX-induced changes in adiposity and insulin resistance. J. Endocrinol..

[40] Rector R.S., Thyfault J.P., Morris R.T., Laye M.J., Borengasser S.J., Booth F.W., Ibdah J.A. (2008). Daily exercise increases hepatic fatty acid oxidation and prevents steatosis in Otsuka Long-Evans Tokushima Fatty rats. Am. J. Physiol. Gastrointest. Liver Physiol..

[41] Santos R.S., de Fatima L.A., Frank A.P., Carneiro E.M., Clegg D.J. (2017). The effects of 17 alpha-estradiol to inhibit inflammation in vitro. Biol. Sex. Differ..

[42] Vieira-Potter V.J., Mishra G., Townsend K.L. (2026). Health of adipose tissue: Oestrogen matters. Nat. Rev. Endocrinol..

[43] Hinton P.S., Ortinau L.C., Dirkes R.K., Shaw E.L., Richard M.W., Zidon T.Z., Britton S.L., Koch L.G., Vieira-Potter V.J. (2018). Soy protein improves tibial whole-bone and tissue-level biomechanical properties in ovariectomized and ovary-intact, low-fit female rats. Bone Rep..

[44] Yamauchi T., Kamon J., Waki H., Terauchi Y., Kubota N., Hara K., Mori Y., Ide T., Murakami K., Tsuboyama-Kasaoka N. (2001). The fat-derived hormone adiponectin reverses insulin resistance associated with both lipoatrophy and obesity. Nat. Med..

[45] Foryst-Ludwig A., Clemenz M., Hohmann S., Hartge M., Sprang C., Frost N., Krikov M., Bhanot S., Barros R., Morani A. (2008). Metabolic actions of estrogen receptor beta (ERbeta) are mediated by a negative cross-talk with PPARgamma. PLoS Genet..

[46] Yang S.H., Liu R., Perez E.J., Wen Y., Stevens S.M., Valencia T., Brun-Zinkernagel A.M., Prokai L., Will Y., Dykens J. (2004). Mitochondrial localization of estrogen receptor beta. Proc. Natl. Acad. Sci. USA.

[47] Yager J.D., Chen J.Q. (2007). Mitochondrial estrogen receptors—New insights into specific functions. Trends Endocrinol. Metab..

[48] Fuentes N., Silveyra P. (2019). Estrogen receptor signaling mechanisms. Adv. Protein Chem. Struct. Biol..

[49] Lephart E.D., Thompson J.M., Setchell K.D., Adlercreutz H., Weber K.S. (2000). Phytoestrogens decrease brain calcium-binding proteins but do not alter hypothalamic androgen metabolizing enzymes in adult male rats. Brain Res..

[50] Correa S.M., Newstrom D.W., Warne J.P., Flandin P., Cheung C.C., Lin-Moore A.T., Pierce A.A., Xu A.W., Rubenstein J.L., Ingraham H.A. (2015). An estrogen-responsive module in the ventromedial hypothalamus selectively drives sex-specific activity in females. Cell Rep..

[51] Vieira-Potter V.J. (2014). Inflammation and macrophage modulation in adipose tissues. Cell. Microbiol..

[52] Choi J.S., Koh I.U., Song J. (2012). Genistein reduced insulin resistance index through modulating lipid metabolism in ovariectomized rats. Nutr. Res..

[53] Sakamoto Y., Naka A., Ohara N., Kondo K., Iida K. (2014). Daidzein regulates proinflammatory adipokines thereby improving obesity-related inflammation through PPARgamma. Mol. Nutr. Food Res..

[54] Sakamoto Y., Kanatsu J., Toh M., Naka A., Kondo K., Iida K. (2016). The Dietary Isoflavone Daidzein Reduces Expression of Pro-Inflammatory Genes through PPARalpha/gamma and JNK Pathways in Adipocyte and Macrophage Co-Cultures. PLoS ONE.

[55] Gregorio K.C.R., Laurindo C.P., Freitas H.S., Okamoto M.M., Seraphim P.M., Machado U.F. (2025). Biphasic Slc2a4 Gene Expression in 3T3-L1 Adipocytes in Response to Treatment with Low and High Concentrations of Daidzein and Genistein. Curr. Issues Mol. Biol..

